# Gut microbiome features associated with vancomycin-resistant *Enterococcus* acquisition in intensive care unit patients

**DOI:** 10.1186/s12941-026-00871-6

**Published:** 2026-06-02

**Authors:** Yu-Chung Chuang, Ronald G. Collman, Sheng-Yuan Ruan, Ying-Chun Chien, Yu-Shan Huang, Cheng-Chih Hsu, Jann-Tay Wang, Hsin-Bai Zou, Shan-Chwen Chang

**Affiliations:** 1 Department of Internal Medicine, National Taiwan University Hospital, 7 Chung-Shan South Road, Taipei, 100226 Taiwan; 2 Department of Microbiology, Perelman School of Medicine, University of Pennsylvania, Philadelphia, PA USA; 3 Division of Pulmonary, Allergy, and Critical Care, Department of Medicine, Perelman School of Medicine, University of Pennsylvania, Philadelphia, PA USA; 4 Department of Chemistry, National Taiwan University, Taipei, Taiwan; 5 Leeuwenhoek Laboratory Co., Ltd, Taipei, Taiwan

**Keywords:** Microbiome, Bile acids, Vancomycin-resistant enterococci, Colonization, *Clostridium scindens*

## Abstract

**Background:**

Vancomycin-resistant *Enterococcus* (VRE) infection poses a significant healthcare burden in intensive care units (ICUs), and is preceded by gut colonization. The gut microbiome may influence susceptibility to VRE, but its role in ICU patients remains incompletely defined.

**Methods:**

We conducted a prospective study of patients admitted to a medical ICU from 2019 to 2021. Stool samples were collected for bacterial 16 S rRNA gene sequencing, anal swabs were screened for VRE by culture, and bile acids were measured in initial stool samples.

**Results:**

We enrolled 108 patients. Thirty-four patients were VRE + on initial screen and remained so (VRE+/+) while 74 were initially negative, of whom 23 acquired VRE (VRE-/+) and 51 remained negative (VRE-/-). There was no difference in alpha-diversity initially between VRE-/- and VRE-/+ groups, whereas VRE+/+ patients had significantly lower alpha-diversity (*P* < 0.001). VRE-/+ patients had a significantly more rapid decrease in alpha-diversity than VRE-/- patients (*P* = 0.04). Beta-diversity of initial stool differed among groups (*P* = 0.001), driven mainly by VRE+/+ patients. A lower *Bacteroides/Enterococcus* ratio (*P* = 0.049) and low *Clostridium scindens* abundance (*P* = 0.031) were associated with VRE acquisition. Initial stool from VRE-/- patients had a higher combined concentration of deoxycholic acid and lithocholic acid than that of VRE-/+ patients (*P* = 0.034).

**Conclusions:**

VRE acquisition in the ICU was associated with an initial gut microbiome characterized by lower *Bacteroides*/*Enterococcus* ratios, lower *C. scindens* abundance, and lower deoxycholic and lithocholic acid concentrations. Our findings are consistent with a possible role of these microbiome features in colonization resistance, as suggested by in vitro and animal models. However, given the single-center design, these associations should be considered hypothesis-generating and require validation before clinical application.

**Supplementary Information:**

The online version contains supplementary material available at 10.1186/s12941-026-00871-6.

## Introduction

Multiple drug-resistant bacteria are substantial threats worldwide [1]. Antimicrobial resistance causes high morbidity, mortality, and healthcare burdens, especially in intensive care units (ICU) [2]. Vancomycin-resistant *Enterococcus* (VRE), first identified in 1986 [3] has become a major cause of healthcare-associated infections [1, 2], including in ICU settings [4].

Microbiome disruption with loss of colonization resistance may permit intestinal expansion of VRE, which in some high-risk settings has been associated with subsequent invasive infection [5]. Colonization resistance is a key function of the normal gut microbiome, and fecal transplants have been considered as a means to eradicate VRE colonization [6]. Gut microbiota might prevent VRE colonization via stimulating innate immunity [7] or direct antagonism [8, 9]. While the mechanisms of antagonisms remain poorly defined, the secondary bile acid deoxycholic acid (DCA) has been correlated with eubiosis and exhibits dose-dependent inhibition of *E. faecalis* [10]. Both DCA and lithocholic acid (LCA) inhibit the growth of VRE in vitro [11]. *Clostridum scindens* has been suggested to contribute to resistance against VRE colonization, possibly through biosynthesis of secondary bile acids [12]. Obligate anaerobic commensal bacteria have been associated with colonization resistance to VRE in murine models [8]. *Barnesiella* spp. was associated with decreased risk of VRE dominance in patients following allo-hematopoietic stem cell transplantation (HSCT) [13]. However, to date no gut microbiome features have been associated with VRE colonization in humans [14], and the factors responsible for VRE colonization resistance remain undefined.

We hypothesized that specific gut microbiome features might be associated with acquisition of VRE colonization. Additionally, we aimed to investigate the association between VRE colonization and bile acids, particularly DCA and LCA. Because ICU admission is a common risk for VRE colonization and infection [15], we conducted a prospective observational study in a medical ICU to delineate gut microbiome features associated with the acquisition of VRE colonization.

## Methods

### Hospital setting and design

This was a prospective observational study conducted at National Taiwan University Hospital, Taipei, Taiwan. Patients were enrolled between January 2020 and October 2021. No patient contributed more than one ICU admission to the analytic cohort. The Research Ethics Committee approved the study and patients were enrolled following written informed consent (201811008RIND). The study was conducted in a medical ICU that primarily admits patients with community-onset acute critical illness. The ICU has single-bed rooms with an anteroom design. Patients were screened for VRE at ICU admission, and contact isolation was instituted once VRE was identified.

Patients had an anal swab for VRE screening on the day of enrollment and then twice weekly till ICU discharge. Stool samples were collected on the same day as the anal swab if available. Samples were frozen at -80 ℃ till use. Healthy controls were selected from metagenomic data available in the Sequence Read Archive of NCBI (https://www.ncbi.nlm.nih.gov/sra/PRJNA628533), which includes samples from healthy outpatients undergoing routine health exams in Taiwan [16]. To reduce age-related confounding of gut microbiome composition, we restricted the comparison to adults aged 35 years or older.

### Stool DNA extraction, 16 S rRNA gene sequencing, and bioinformatics analysis

Fecal DNA was extracted using QIAamp PowerFecal Pro DNA Kit (QIAGEN, Hilden, Germany). The 16 S gene V3-V4 region was amplified (detailed in the supplement). Sequences are deposited in the National Center for Biotechnology Information.

Sequence Read Archive under PRJNA1033627. Reads were imported to QIIME2 (version 2021.8) [17], denoised and merged using DADA2, and clustered into Amplicon Sequence Variants (ASV). The SILVA rRNA database (release 138) was used for taxonomy assignment of clustered ASVs. The ASV table generated by QIIME2 was used for calculating alpha-diversity with the Shannon index, Berger-Parker index of dominance, and beta-diversity using the weighted UniFrac distance matrix. The UniFrac distance matrices were used to perform principal coordinate analysis (PCoA) to compare bacterial composition between sample groups. PICRUSt2 was used for prediction of metagenome functions [18]. Oxygen utilization of bacteria was classified using BugBase [19]. The software unassigner as also used to confirm the bacterial taxonomy classification of the ASV [20].

### Bile acids analysis

The concentrations of DCA and LCA in the stool samples were measured using Ultra-high-performance liquid chromatography–tandem mass spectrometry (detailed in the supplement).

### Clinical data collection and definitions

Clinical characteristics were extracted from the electronic medical record, including past admission history, underlying disease, antibiotic exposure at ICU admission, and antibiotic exposure during ICU stay before VRE acquisition or censoring. Patients’ underlying comorbidities were evaluated using the Charlson comorbidity index [21]. Antimicrobial agents were categorized into anti-anaerobics [22], glycopeptides, and carbapenems (detailed in the supplement).

VRE screening was performed using chromID VRE (bioMérieux, Marcy l’Étoile, France). Patients with VRE on initial screen all remained positive and were classified as VRE+/+. Patients initially VRE negative who converted to VRE positive were classified as VRE-/+. Patients without VRE on initial screen who remained negative were classified as VRE-/-. Because stool was not always available, some VRE-/+ patients did not have samples available before VRE colonization. Thus, the Entire Cohort (EC) included all enrolled patients, whereas the Predictive Cohort (PC) excluded VRE-/+ patients without available stool samples prior to VRE colonization (Table S1 in the online data supplement).

### Statistical analysis

Alpha-diversity (Shannon index) was compared using Wilcoxon rank-sum test. Beta-diversity was compared using permutational multivariate analysis of variance (PERMANOVA). The compositions of microbiomes and predicted metagenome functions were compared using analysis of compositions of microbiomes with bias correction (ANCOM-BC) [23]. For clinical features, continuous variables were compared using Wilcoxon rank-sum test; categorical variables were compared using Fisher’s exact test. The longitudinal follow-up of Shannon diversity among different groups was compared using generalized estimating equations (GEE). The statistical analysis was performed using R version 4.1.1. (R Foundation for Statistical Computing, Vienna, Austria).

### Use of artificial intelligence tools

During manuscript preparation, we used ChatGPT (GPT-4.0, OpenAI) to assist with English language editing. All content was subsequently checked and approved by the authors, who take full responsibility for the manuscript.

## Results

### Subjects and clinical features

We enrolled 108 patients admitted to the ICU. Baseline characteristics are summarized in Table 1. Thirty-four patients were VRE + on initial screen and remained so (VRE+/+), 74 were initially negative, of whom 23 converted to positive (VRE-/+), and 51 remained negative (VRE-/-). Among the 23 patients who were VRE-negative at ICU admission and subsequently acquired VRE colonization, the median time from ICU admission to first VRE-positive screen was 6 days (IQR 3–8). Antibiotic exposure during ICU stay prior to VRE acquisition or censoring did not differ significantly between the VRE -/- and VRE -/+ groups (Table S2).

**Table 1:** Clinical characteristics of the entire cohort (EC)

Variable^a^	VRE-/- (*n* = 51)	VRE-/+ (*n* = 23)	VRE+/+ (*n* = 34)	*P* ^b^	*P* ^c^
Demographics					
Age (years)	71.7 (58.3–82.4)	73.2 (62.0–81.1)	76.8 (59.9– 85.1)	0.62	0.92
Male	26 (51.0)	13 (56.5)	23 (67.6)	0.30	0.80
Hospital stay ≤ 3 days before ICU admission	39 (76.5)	19 (82.6)	26 (76.5)	0.87	0.76
Duration to first sample (days)	3 (2–4)	4 (2–7)	3 (2–6)	0.06	0.02
Nursing home residents	1 (2.0)	0 (0)	2 (5.9)	0.45	0.99
Admission history within 3 months	17 (33.3)	9 (39.1)	23 (67.6)	0.007	0.79
Underlying condition					
Charlson Comorbidity Index	3 (2–6)	3 (2–6)	4 (3–6)	0.50	0.63
Myocardial infarction	1 (2.0)	0 (0)	1 (2.9)	0.99	0.99
Congestive heart failure	16 (31.4)	8 (34.8)	15 (44.1)	0.48	0.79
Cerebrovascular disease	2 (3.9)	0 (0)	3 (8.8)	0.43	0.99
Chronic obstructive pulmonary disease	1 (2.0)	2 (8.7)	5 (14.7)	0.06	0.23
Autoimmune disease	5 (9.8)	0 (0)	2 (5.9)	0.36	0.32
Liver cirrhosis	5 (9.8)	3 (13.0)	2 (5.9)	0.57	0.70
Diabetes mellitus	12 (23.5)	9 (39.1)	8 (23.5)	0.34	0.18
Chronic kidney disease	14 (27.4)	5 (21.7)	11 (32.4)	0.70	0.78
Renal replacement therapy	9 (17.6)	3 (13.0)	8 (23.5)	0.62	0.74
Malignancy	20 (39.2)	14 (60.9)	14 (41.2)	0.21	0.13
Hematologic malignancy	4 (7.8)	3 (13.0)	6 (17.6)	0.38	0.67
Use of immunosuppressive agents	31 (60.8)	20 (87.0)	26 (76.5)	0.04	0.03
Corticosteroids	30 (58.8)	20 (87.0)	26 (76.5)	0.04	0.02
Chemotherapy	8 (15.7)	3 (13.0)	8 (23.5)	0.61	0.99
Initial antibiotics at intensive care unit admission					
Carbapenems	11 (21.6)	4 (17.4)	12 (35.3)	0.27	0.76
Anti-Pseudomonas Carbapenems	11 (21.6)	3 (13.0)	11 (32.4)	0.24	0.53
Anti-Anaerobics	40 (78.4)	21 (91.3)	30 (88.2)	0.35	0.32
Glycopeptide	9 (17.6)	8 (34.8)	12 (35.3)	0.12	0.14
Duration of ICU stay	8 (6–13)	15 (11–22)	12 (7–19)	0.003	0.001
In-hospital mortality	24 (47.1)	11 (47.8)	18 (52.9)	0.88	0.99

^*a*^For continuous variables, median and interquartile range were presented and compared using either the Kruskal-Wallis test for three or more groups or the Wilcoxon rank-sum test for two groups. Categorical variables were presented as numbers and percentages and compared using Fisher’s exact test

^*b*^comparisons among three groups

^*c*^comparison between the VRE-/- and VRE-/+ groups

*VRE-/-* VRE negative on initial screening and remained negative, *VRE-/+* VRE negative on initial screening and acquired VRE during ICU stay, *VRE+/+* VRE positive on initial screening and remained positive, *ICU* intensive care unit

### Gut microbiome features in relation to VRE presence

**Fig. 1:**
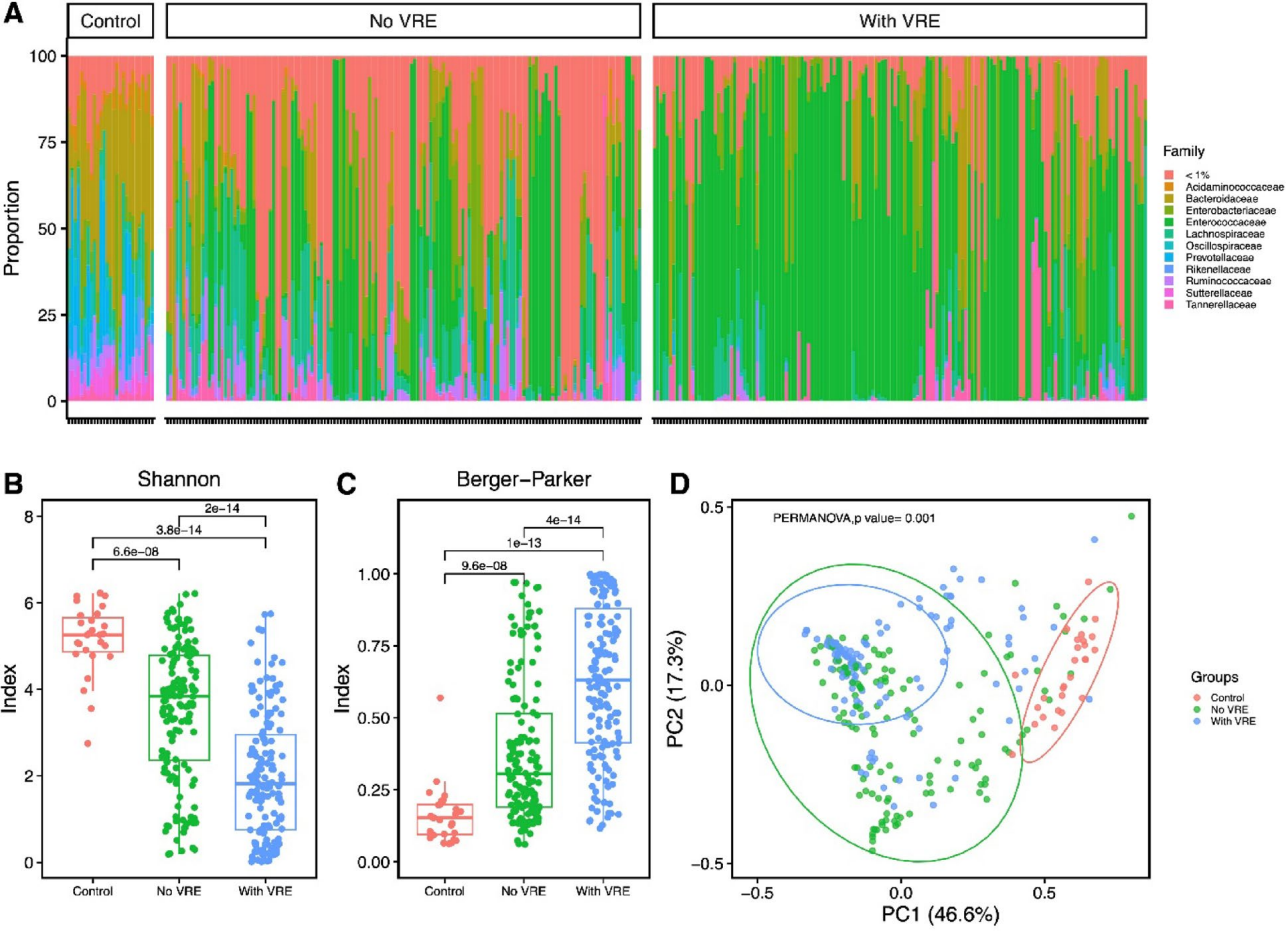
Gut microbiome composition among ICU patient and control samples. **A** Comparison of the composition at the family level among the control samples and ICU subject samples with or without VRE colonization. Samples with VRE colonization were associated with a higher Enterococcaceae relative abundance (*P* = 2e-16) . **B** Comparison of the Shannon diversity index among the control group and ICU subject samples with or without VRE colonization. The control group had a significantly higher Shannon diversity than samples without VRE colonization (*P* = 6.6e-08) and samples with VRE colonization (*P* = 3.8e-14). Samples without VRE colonization had a significant higher Shannon diversity compared to samples with VRE colonization (*P* = 2.0e-14). **C** Comparison of the Berger-Parker dominance index among the control group and ICU subject samples with or without VRE colonization. The Control group had a significantly lower dominance levels than samples without VRE colonization (*P* = 9.6e-08) and samples with VRE colonization (*P* = 1e-13). Samples without VRE colonization had a significant dominance compared to samples with VRE colonization (*P* = 4e-14). **D** Comparison of the beta diversity among the control group and ICU subject samples with or without VRE colonization. The three groups had a significant beta diversity difference (*P* = 0.001). There was a significant beta diversity difference between control groups and samples without VRE colonization (*P* = 0.001) and samples with VRE colonization (*P* = 0.001). There was also a significant beta diversity difference compared between samples without VRE colonization and samples with VRE colonization (*P* = 0.001). *ICU* intensive care unit, *VRE* vancomycin-resistant *Enterococcus*

A total of 302 stool samples from ICU patients were available for 16 S rRNA gene microbiome analysis, among which 154 samples were associated with contemporaneous positive VRE colonization screening and 148 samples were VRE negative. In addition, 27 stool samples from healthy volunteers were available as controls (Fig. 1). Patient samples exhibited higher *Enterococcaceae* relative abundance than control samples (*P* < 2.2e-16). ICU patient samples had significant lower Shannon diversity and higher Berger-Parker dominance index than controls (both *P* < 2.2e-16). Beta diversity was also significantly different among samples with VRE colonization, without VRE colonization, and controls (*P* = 0.001).

### Microbiome features by ICU subject group and change over time

**Fig. 2:**
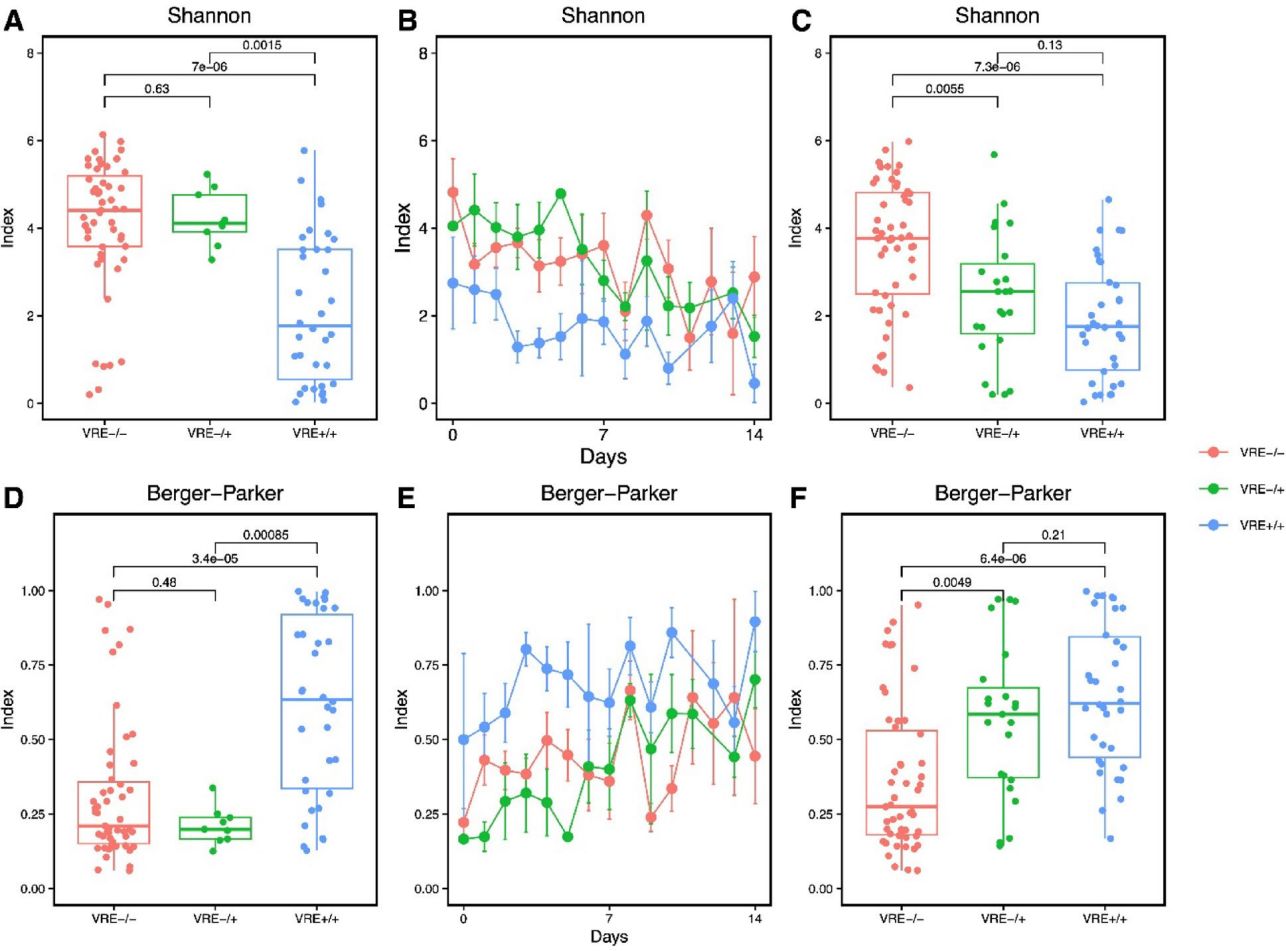
Gut microbiome composition among ICU subjects by VRE colonization group and over time. **A** Shannon diversity index of the first stool sample among the three ICU subject groups. The first sample from VRE-/- and VRE-/+ groups had a significantly higher Shannon diversity than VRE+/+ (*P* = 7e-06 and *P* = 0.0015, respectively). There was no significant Shannon diversity difference between VRE-/- and VRE -/+ groups (*P* = 0.63). **B** The Shannon diversity index changes over time during the first 14 days of intensive care unit follow-up. **C** Shannon diversity index of the last stool sample among the three groups. The last sample from VRE+/+ and VRE-/+ groups had a significantly lower Shannon diversity than VRE-/- (*P* = 7.3e-06 and *P* = 0.0055, respectively). There was no significant Shannon diversity difference between VRE+/+ and VRE -/+ groups (*P* = 0.13). **D** Berger-Parker dominance index of the first stool sample among the three ICU subject groups. The first sample of VRE-/- and VRE-/+ groups had a significantly lower Berger-Parker dominance index than VRE+/+ (*P* = 3.4e-05 and *P* = 0.00085, respectively). There was no significant Berger-Parker dominance index difference between VRE-/- and VRE -/+ groups (*P* = 0.48). **E** The Berger-Parker dominance index changes over time during the first 14 days of intensive care unit follow-up. **F** Berger-Parker dominance index of the last stool sample among the three groups. VRE+/+ and VRE-/+ groups had a significantly higher Berger-Parker dominance index than VRE-/- (*P* = 6.4e-06 and *P* = 0.0049, respectively). There was no significant Berger-Parker dominance index difference between VRE+/+ and VRE -/+ groups (*P* = 0.21). *VRE-/-* VRE negative on initial screening and remained negative, *VRE-/+* VRE negative on initial screening and acquired VRE during ICU stay, *VRE+/+* VRE positive on initial screening and remained positive, *ICU* intensive care unit, *VRE* vancomycin-resistant *Enterococcus*

Characteristics of patients in the predictive cohort (PC; which excludes VRE-/+ subjects if they did not have stool available prior to VRE acquisition) are summarized in Table 2. Shannon diversity is depicted in Fig. 2A-C and the results of modeling of Shannon diversity index using GEE are shown in Table 3.

**Table 2:** Clinical characteristics of the predictive cohort (PC)

Variable^a^	VRE-/- (*n* = 51)	VRE-/+ (*n* = 9)	VRE+/+ (*n* = 34)	*P* ^b^	*P* ^c^
Demographics					
Age (years)	71.7 (58.3–82.4)	66.0 (60.1–79.5)	76.8 (59.9– 85.1)	0.59	0.88
Male	26 (51.0)	7 (77.8)	23 (67.6)	0.17	0.17
Hospital stay ≤ 3 days before ICU admission	39 (76.5)	7 (77.8)	26 (76.5)	0.99	0.99
Duration to first sample (days)	3 (2–4)	3 (1–3)	3 (2–6)	0.50	0.82
Nursing home residents	1 (2.0)	0 (0)	2 (5.9)	0.68	0.99
Admission history within 3 months	17 (33.3)	2 (22.2)	23 (67.6)	0.003	0.71
Underlying condition					
Charlson Comorbidity Index	3 (2–6)	3 (3–6)	4 (3–6)	0.35	0.24
Myocardial infarction	1 (2.0)	0 (0)	1 (2.9)	0.99	0.99
Congestive heart failure	16 (31.4)	4 (44.4)	15 (44.1)	0.40	0.46
Cerebrovascular disease	2 (3.9)	0 (0)	3 (8.8)	0.63	0.99
Chronic obstructive pulmonary disease	1 (2.0)	1 (11.1)	5 (14.7)	0.07	0.28
Autoimmune disease	5 (9.8)	0 (0)	2 (5.9)	0.85	0.99
Liver cirrhosis	5 (9.8)	0 (0)	2 (5.9)	0.85	0.99
Diabetes mellitus	12 (23.5)	4 (44.4)	8 (23.5)	0.44	0.23
Chronic kidney disease	14 (27.4)	3 (33.3)	11 (32.4)	0.80	0.70
Renal replacement therapy	9 (17.6)	1 (11.1)	8 (23.5)	0.73	0.99
Malignancy	20 (39.2)	7 (77.8)	14 (41.2)	0.11	0.07
Hematologic malignancy	4 (7.8)	2 (22.2)	6 (17.6)	0.21	0.22
Use of immunosuppressive agents	31 (60.8)	8 (88.9)	26 (76.5)	0.10	0.14
Corticosteroids	30 (58.8)	8 (88.9)	26 (76.5)	0.11	0.14
Chemotherapy	8 (15.7)	1 (11.1)	8 (23.5)	0.61	0.99
Initial antibiotics at intensive care unit admission					
Carbapenems	11 (21.6)	3 (33.3)	12 (35.3)	0.31	0.42
Anti-Pseudomonas Carbapenems	11 (21.6)	2 (22.2)	11 (32.4)	0.50	0.99
Anti-Anaerobics	40 (78.4)	8 (88.9)	30 (88.2)	0.55	0.67
Glycopeptide	9 (17.6)	3 (33.3)	12 (35.3)	0.16	0.37
Bacterial composition of initial stool sample					
* Enterococcus*/*Bacteroides* ratio	7.78e-01 (1.24e-02–1.08e+01)	4.68e-03 (8.27e-04–1.91e-02)	1.90e-04 (1.54e-05–2.69e-01)	<0.001	0.048
* Bacteroides*/*Enterococcus* ratio < 0.02	14 (27.5)	7 (77.8)	22 (64.7)	<0.001	0.006
* C. scindens* relative abundance (%)	1.41e-02 (0–3.04e-01)	0 (0–0)	0 (0–0)	0.007	0.031
No *C. scindens*	24 (47.1)	7 (77.8)	26 (76.5)	0.013	0.15
* C. scindens* relative abundance ≤0.01%	24 (47.1)	9 (100)	27 (79.4)	<0.001	0.003
Duration of ICU stay	8 (6–13)	14 (12–22)	12 (7–19)	0.005	0.002
In-hospital mortality	24 (47.1)	6 (66.7)	18 (52.9)	0.59	0.47

^*a*^For continuous variables, median and interquartile range were presented and compared using either the Kruskal-Wallis test for three or more groups or the Wilcoxon rank-sum test for two groups. Categorical variables were presented as numbers and percentages and compared using Fisher’s exact test

^*b*^comparisons among three groups

^*c*^comparison between the VRE-/- and VRE-/+ groups

*VRE-/-* VRE negative on initial screening and remained negative, *VRE-/+* VRE negative on initial screening and acquired VRE during ICU stay, *VRE+/+* VRE positive on initial screening and remained positive, *ICU* intensive care unit

**Table 3:** Generalized estimating equation model of Shannon diversity index over the first two weeks in the entire cohort (EC)

Variables	β-coefficient	95% confidence interval	*P*
Initial Shannon diversity index	0.75	0.65 – 0.84	<0.001
Days in ICU	-0.07	-0.11 – -0.02	0.002
VRE groups (reference)	VRE -/- as reference		
VRE-/+	0.44	-0.17 – 1.06	0.16
VRE+/+	-0.53	-1.05 – -0.02	0.04
Interaction term of VRE groups and Days in ICU	VRE -/- as reference		
VRE-/+	-0.08	-0.15 – -0.003	0.04
VRE+/+	0.04	-0.03 – 0.10	0.31
Constant	1.05	0.56 – 1.53	<0.001

*VRE-/-* VRE negative on initial screening and remained negative, *VRE-/+* VRE negative on initial screening and acquired VRE during ICU stay, *VRE+/+* VRE positive on initial screening and remained positive, *ICU* intensive care unit

Within the PC, initial samples of VRE-/- and VRE -/+ groups did not differ in Shannon diversity, while those initially colonized (VRE+/+) were significantly lower than VRE-/- and VRE -/+ groups (Fig. 2A; *P* = 7e-06 and *P* = 0.0015, respectively).

On average among all patients, the Shannon diversity decreased by 0.07 per day (*P* = 0.002). At each time point, the VRE+/+ group had a significantly lower Shannon diversity than the VRE-/- group (-0.53; *P* = 0.04), and the slope of decrease was not significantly different between the VRE-/- and VRE+/+ groups (Table 3; Fig. 2B). However, the VRE-/+ group had a significantly more rapid decrease in Shannon diversity than VRE-/- group (slope difference of -0.08/day; *P* = 0.04). As a result, the last samples of VRE-/+ patients were not different from the VRE +/+ groups in Shannon diversity, whereas the VRE-/- group was significantly higher than both VRE-/+ and VRE +/+ (Fig. 2C; *P* = 0.0055 and *P* = 7.3e-06, respectively).

An inverse pattern was seen with the Berger-Parker dominance index (Fig. 2D-F). Thus, patients who acquire VRE during ICU stay start out resembling those who began and remained VRE-free, but exhibit a more rapid decrease in diversity and increase in dominance, and end up resembling patients who initially presented with and remained VRE-colonized.

### Initial stool features that distinguish patients who do or do not acquire VRE in the ICU

**Fig. 3:**
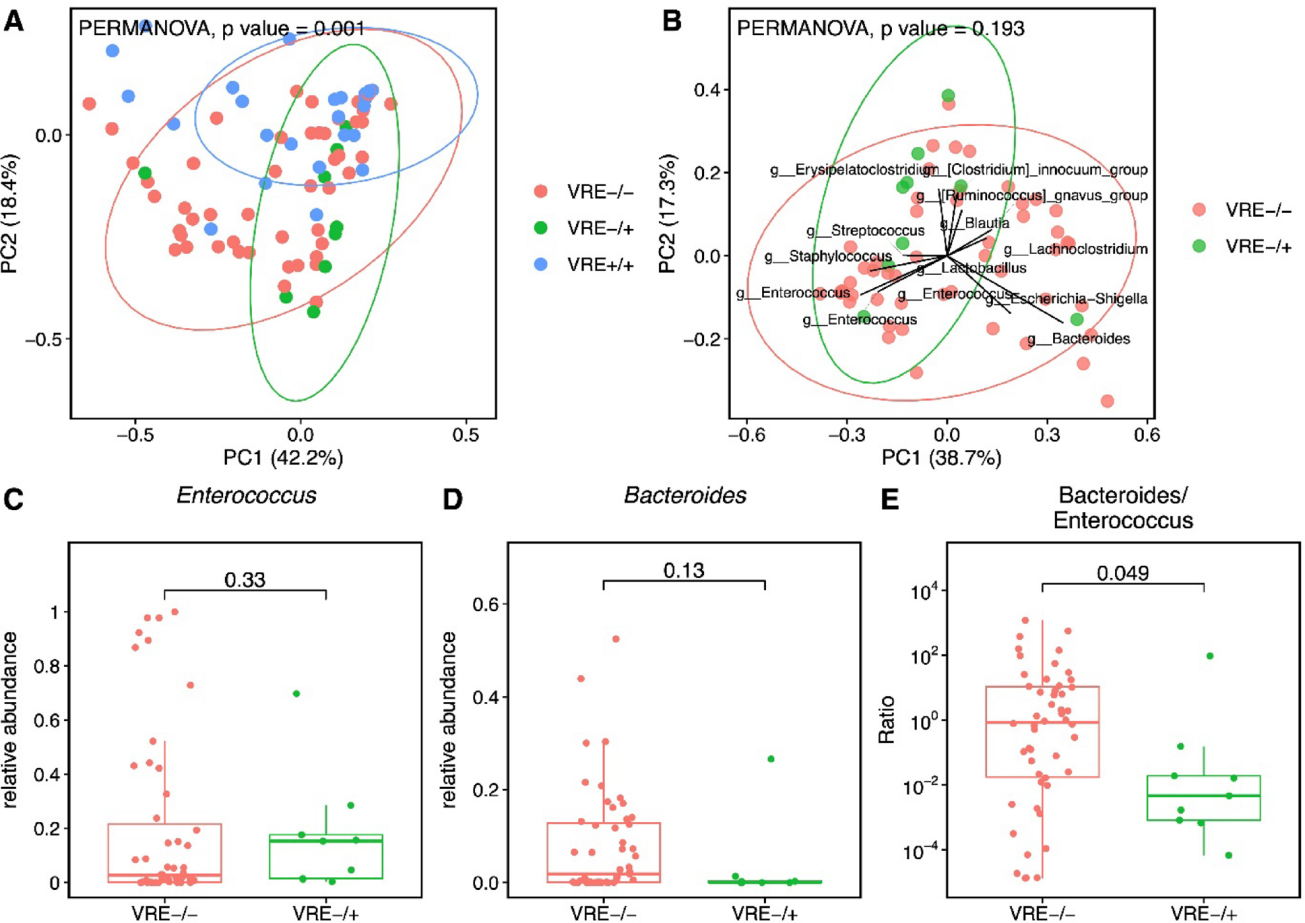
Initial microbiome features by subject group in the predictive cohort. **A** Beta diversity of the first stool sample among the three groups was significantly different (*P* = 0.001). Individual group comparisons showed significant differences between VRE-/- and VRE+/+ (*P* = 0.001) and VRE-/+ and VRE +/+ (*P* = 0.005). **B** Beta diversity comparing the first stool sample of VRE-/- and VRE -/+ groups was not significantly different (*P* = 0.193). However, dispersion was seen along PC1, which was substantially driven by *Bacteroides* spp. to the right and *Enterococcus* spp. to the left. **C** There was no significant difference in the relative abundance of *Enterococcus* spp. (*P* = 0.33 compared between the VRE-/- and VRE-/+ groups. **D** There was no significant difference in the relative abundance of *Bacteroides* spp. (*P* = 0.13) compared between the VRE-/- and VRE-/+ groups. **E** The first stool samples of the VRE-/- group had a significantly higher ratio of *Bacteroides* spp. to *Enterococcus* spp. compared to the VRE-/+ group (*P* = 0.049). *VRE-/-* VRE negative on initial screening and remained negative, *VRE-/+* VRE negative on initial screening and acquired VRE during ICU stay, *VRE+/+* VRE positive on initial screening and remained positive, *ICU* intensive care unit, *VRE* vancomycin-resistant *Enterococcus*

Within the PC, global composition of the first stool sample based on weighted UniFrac was significantly different among the three groups (*P* = 0.002), especially comparing between VRE-/- and VRE+/+ (*P* = 0.001) and VRE-/+ and VRE+/+ (*P* = 0.005) (Fig. 3A). However, the initial stool community by weighted UniFrac was not significant different between VRE-/- and VRE-/+ (*P* = 0.193) (Fig. 3B).

### Taxa associated with VRE acquisition

As shown in Fig. 3B, in the initial stool samples of the PC, *Bacteroides* spp. appeared to contribute PCoA loadings toward VRE-/- samples and *Enterococcus* spp. contributed to the direction toward VRE-/+ (Fig. 3B). Therefore, we examined the relative abundance of these taxa. Although relative abundances of *Enterococcus* and *Bacteroides* spp. in the initial stool were not significantly different between the VRE-/- and VRE-/+ groups (*P* = 0.33 and *P* = 0.13, respectively), there was a significantly higher *Bacteroides/Enterococcus* ratio in the initial stool of those who remained VRE-free (*P* = 0.049). In contrast, there was no significant difference in the relative abundance of anaerobic bacteria overall (classified according to the BugBase database) between the VRE -/- and VRE-/+ groups (*P* = 0.8).

**Fig. 4:**
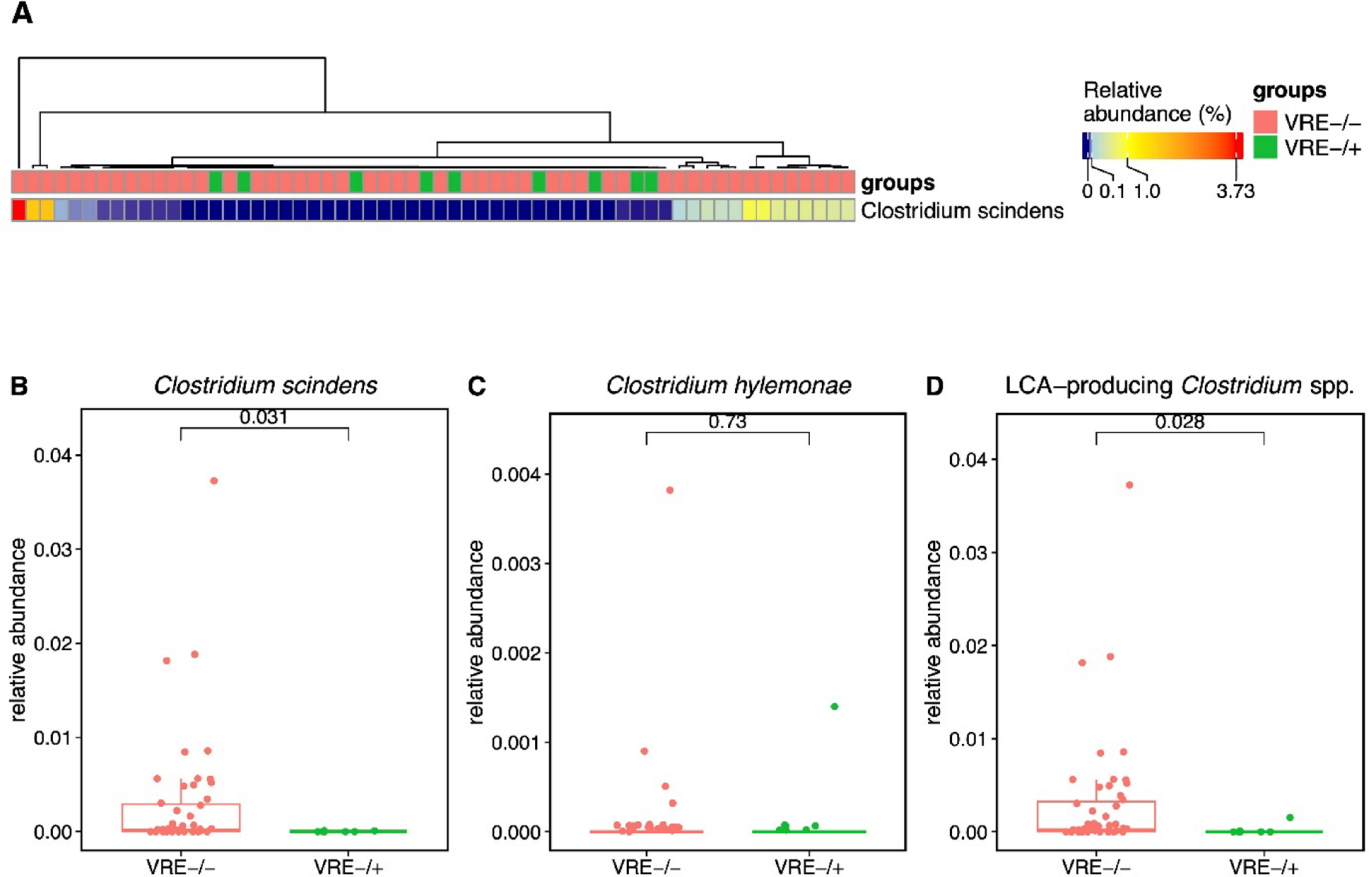
Taxa associated with subsequent acquisition of VRE colonization. **A** Analysis using ANCOM-BC showed that *C. scindens* in the first stool sample is inversely associated with subsequent VRE acquisition. **B** Among the lithocholic acid (LCA) producing Clostridium spp, lack of VRE acquisition was associated with a higher relative abundance of *C. scindens* in the first stool sample (*P* = 0.031; Wilcoxon rank sum) but not C. *hylemonae*. *VRE-/-* VRE negative on initial screening and remained negative, *VRE-/+* VRE negative on initial screening and acquired VRE during ICU stay, *LCA* lithocholic acid, *ICU* intensive care unit, *VRE* vancomycin-resistant *Enterococcus*

We then investigated taxa within the initial stool of VRE-/- and VRE-/+ subjects in the PC using ANCOM-BC, which performs differential abundance and correlation analysis with bias correction. This revealed a significant difference in abundance of *C. scindens* (ANCOM-BC FDR-adjusted *P* = 0.028) between initial sample of VRE-/- and VRE-/+ patients (Fig. 4A).

Because *C. scindens* is known to produce LCA, which has been reported to impede VRE growth [11], we queried other LCA-producing bacteria in the initial stool sample including *C. hylemonae*, and *C. hiranosis* (Fig. 4B). There was no significant association with *C. hylemonae* (*P* = 0.73), and *C. hiranosis* was not detected in the samples, but *C. scindens* abundance remained significantly associated with subsequent VRE-free status in this analysis (*P* = 0.031).

### Imputed metagenomic features associated with VRE acquisition

We then applied PICRUSt2 to predict metagenomic functions associated with sustained VRE-free status. The bile acid-inducible (*Bai*) operon, which support secondary bile acid synthesis, was identified from the KEGG Orthology database [24]. We therefore queried the predicted *Bai* operon using ANCOM-BC (Table 4). Among pathways involved in secondary bile acid synthesis, *BaiA* was associated with VRE-/- (beta, -1.54; *P* = 0.04). These findings implicate bile acid metabolism as a possible link between the microbiome and VRE resistance.

**Table 4:** PICRUSt2-predicted bai operon abundance in VRE-/- and VRE-/+ groups in the predictive cohort (PC)

KEGG orthology	Name	β coefficient	Standard error	*P* value	Q value
K15869	*baiA*; 3alpha-hydroxy bile acid-CoA-ester 3-dehydrogenase [EC:1.1.1.395]	-1.54	0.75	0.04	0.27
K15872	*baiE*; bile-acid 7alpha-dehydratase [EC:4.2.1.106]	-1.22	0.71	0.09	0.27
K15871	*baiF*; bile acid CoA-transferase [EC:2.8.3.25]	-1.20	0.76	0.12	0.27
K15868	*baiB*; bile acid-coenzyme A ligase [EC:6.2.1.7]	-0.61	0.73	0.40	0.50
K15873	*baiH*; NAD+-dependent 7beta-hydroxy-3-oxo bile acid-CoA-ester 4-dehydrogenase	1.04	0.86	0.22	0.39
K15874	*baiI*; bile acid 7beta-dehydratase	-0.77	0.97	0.43	0.50
K15870	*baiCD*; NAD+-dependent 7alpha-hydroxy-3-oxo bile acid-CoA-ester 4-dehydrogenase	-0.21	0.70	0.76	0.77

*VRE-/-* VRE negative on initial screening and remained negative, *VRE-/+*, VRE negative on initial screening and acquired VRE during ICU stay

Negative β coefficients indicate lower predicted gene abundance in VRE-/+ compared with VRE

### Association between bile acids and VRE acquisition

**Fig. 5:**
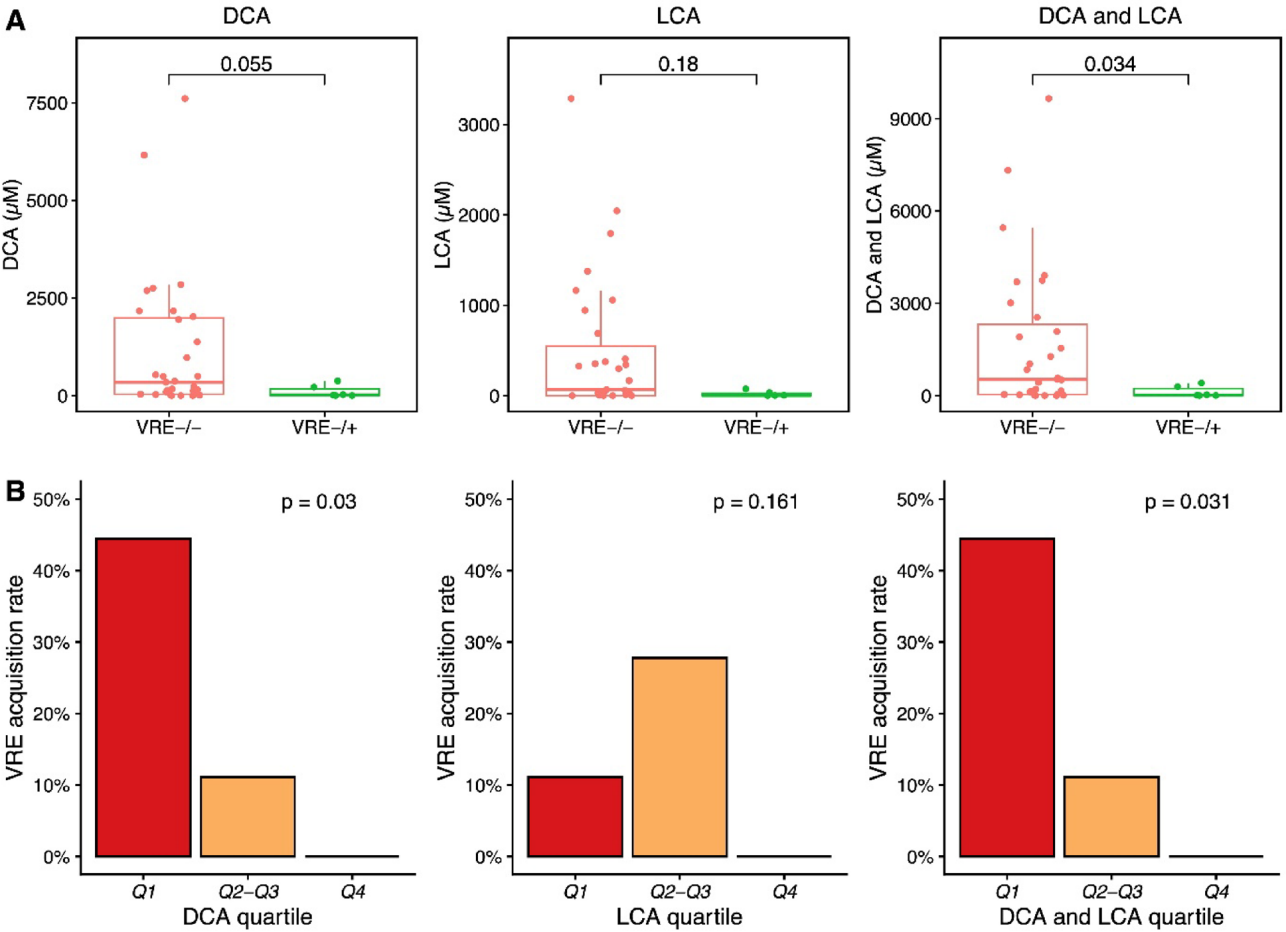
Association between deoxycholic acid (DCA) and lithocholic acid (LCA) concentrations and subsequent VRE acquisition. **A** The combined concentration of DCA and LCA is inversely associated with VRE acquisition. **B** None of the patients in the 4th quartile of DCA and LCA concentrations experienced VRE acquisition. *DCA* deoxycholic acid, *LCA* lithocholic acid, *VRE-/-* VRE negative on initial screening and remained negative, *VRE-/+* VRE negative on initial screening and acquired VRE during ICU stay, *ICU* intensive care unit, *VRE* vancomycin-resistant *Enterococcus*

Among the PC patients, DCA and LCA concentrations were highly correlated (rho = 0.84). Given their shared metabolic pathway and known VRE-inhibitory effects [11], we analyzed their combined concentration. While individual differences did not reach significance (*P* = 0.054 and *P* = 0.18, respectively), combined DCA/LCA levels were significantly higher in VRE-/- patients (*P* = 0.034; Fig. 5A). Notably, patients in the highest quartile for DCA or combined DCA/LCA had significantly lower VRE acquisition (*P* = 0.03 and *P* = 0.031; Fig. 5B).

## Discussion

VRE colonization is a precursor to VRE infection, a major and growing hazard in ICU patients. We found a high rate of VRE colonization on initial screening at ICU admission (31.5%), and among those initially negative, a high VRE acquisition rate (31.1%) despite infection control measures. Among patients admitted to the ICU without VRE colonization, a low initial *Bactericides*/*Enterococcus* ratio was associated with subsequent VRE acquisition. Conversely, initially high *C. scindens* relative abundance, greater representation of imputed metabolic pathways involved in bile acid synthesis, and high levels of the bile acids LCA and DCA were associated with sustained VRE-free status.

VRE colonization at admission is a major determinant of the VRE burden in the ICU [25]. Although there are large variations among studies, a recent meta-analysis reported an average VRE colonization rate at ICU admission of 8.8% (95% CI 7.1–10.6) [25]. Previous studies reported Taiwan’s VRE colonization rate at ICU admission was 3.9–10.9% [26–28] but the present study showed a much higher initial prevalence (31.5%). Though differences in patient populations, hospital settings, or VRE screening methods among studies may be in part responsible, this finding also highlights the magnitude of the increasing VRE burden. The high colonization prevalence at admission might also contribute to the higher in-hospital acquisition rate of the present study (31.1%) versus 10.2% (95% CI 7.7–13) in previous studies [25].

Sequential stool samples showed that alpha-diversity decreased over time after ICU admission, especially in the VRE-/+ group. Importantly, GEE analysis (which included VRE-/-, VRE+/+, and VRE-/+ pre-VRE-colonization; Table 5) showed that patients who acquired VRE had a more rapid decrease in Shannon diversity even prior to VRE acquisition than those who remained VRE-negative (-0.13/day; *P* = 0.037). This finding suggests that decreased diversity preceded VRE acquisition in this cohort; however, because patients who acquired VRE also had longer ICU stays (median 14 vs. 7 days, *P* = 0.001), the potential influence of differential time at risk should be considered. The reason for the more rapid decrease in these patients is unclear. Neither antibiotic exposure at ICU admission nor antibiotic exposure during ICU stay prior to VRE acquisition differed significantly between groups (Table S2), though these comparisons are limited by the small number of acquisition events and the inability to account for time-varying exposure in a multivariable model.

**Table 5:** Generalized estimating equation model of Shannon diversity index over the first two weeks in the entire cohort, including VRE-/+, VRE+/+, and VRE-/- groups (VRE-/+ evaluated only prior to VRE colonization)

Variables	β-coefficient	95% confidence interval	*P*
Initial Shannon diversity index	0.77	0.67 – 0.87	<0.001
Days in ICU	-0.07	-0.11 – -0.03	0.001
VRE groups (reference)	VRE -/- as reference		
VRE-/+^*a*^	0.60	-0.19 – 1.39	0.14
VRE+/+	-0.49	-0.98 – 0.00	0.05
Interaction term of VRE groups and Days in ICU	VRE -/- as reference		
VRE-/+^*a*^	-0.13	-0.26 – -0.008	0.04
VRE+/+	0.03	-0.03 – 0.10	0.29
Constant	0.98	0.48 – 1.47	<0.001

^a^Only stool samples collected before VRE colonization in the VRE-/+ group were included in this model

*VRE-/-* VRE negative on initial screening and remained negative, *VRE-/+* VRE negative on initial screening and acquired VRE during ICU stay, *VRE+/+*, VRE positive on initial screening and remained positive, *ICU* intensive care unit

Obligate anaerobic commensal bacteria have been associated with VRE colonization resistance in mice [8]. We evaluated the impact of obligate anaerobes, including *Bacteroides* spp. While there was no difference in overall obligate anaerobic bacteria, there was numerically higher *Bacteroides* spp. among the VRE−/− group compared to the VRE−/+ group, although this association was not statistically significant. Nevertheless, a higher *Bacteroides*/*Enterococcus* ratio was inversely correlated with VRE acquisition. This finding implies that VRE colonization may occur in the context of an overall *Enterococcus*-favored environments, associated with low *Bacteroides* anaerobes. In addition to direct antagonism, gut microbiota might interact with VRE through innate immunity [29]. Murine models suggest that a healthy gut microbiome community facilitates fucosylation of gut epithelium via IL-22, which expands the *Bacteroides* community, which might further inhibit VRE growth [30]. Additionally, antimicrobial disruption of the gut microbiome may downregulate RegIIIγ, a lectin that kills Gram-positive bacteria including VRE, thus facilitating VRE colonization [7].

In our predictive cohort, the abundance *C. scindens* in initial samples was significantly associated with resistance to VRE colonization. *C. scindens* is known to synthesize the secondary bile acid DCA, and can prevent *C. difficile* infection [31]. Patients with *C. difficile* infection exhibit a low diversity, and typically exhibit high abundances of *Enterococcus* spp [32]. Conversely, among patients with VRE infection or colonization, *C. difficile* is a common cause of co-infection [33]. Thus, similar mechanism may contribute to resistance to both pathogens. It is hypothesized that *C. scindens* may enhance VRE intestinal clearance in mice via secondary bile acids [12], though to our knowledge no prior study has demonstrated a correlation between *C. scindens* and VRE colonization in patients. In addition to DCA, *C. scindens* is also capable of synthesizing LCA [34]. LCA impairs the separation of VRE diplococci, impairing growth [11]. Using ANCOM-BC, we found that lower initial abundance of *C. scindens* was associated with subsequent VRE colonization (*P* = 0.031). In this cohort, no patient with an initial *C. scindens* relative abundance greater than 0.01% acquired VRE colonization during ICU follow-up; however, this observation is based on a small sample and should be interpreted cautiously. Notably, higher concentrations of DCA or a combination of DCA/LCA were significantly associated with lower VRE acquisition. Exploratory imputed metagenomic analysis suggested higher predicted abundance of bile acid 7α-dehydroxylation genes (for example *baiA* and *baiE*) in VRE-/- patients, but these associations did not remain significant after correction for multiple testing and should be interpreted with caution. These results are consistent with the hypothesis that *C. scindens* and related bile acid metabolism may be associated with resistance to VRE acquisition, but this study does not establish causality. Our findings differ from a previous report that did not identify microbiome features linked to VRE acquisition in hospitalized patients [14]. However, that cohort differed from ours in clinical setting and study design. Their study incorporated time-at-risk considerations, whereas our cohort focused on a medical ICU population at high risk of VRE acquisition. These methodological differences may contribute to the differing results [15].

Several other specific taxa have been associated with VRE colonization resistance. *Blautia producta* inhibited VRE growth in vitro and reduced VRE density in animal models [9]. A consortium of *B. producta*, *Clostridium bolteae*, *Bacteroides sartorii*, and *Parabacteroides distasonis* entirely prevented VRE expansion in mice [8]. *Barnesiella* was not only associated with VRE elimination in mice but also diminished the risk of VRE domination in allo-HSCT patients [13]. However, in this ICU cohort we identified no *B. producta* or *C. bolteae*, and higher *Barnesiella* spp. relative abundance was not associated with less VRE acquisition (*P* = 0.81). Thus, multiple mechanisms of VRE resistance are likely to exist, and these may differ between ICU and HSCT populations.

Our study has several important limitations. First, this was a small, single-center study in a specific medical ICU setting, which limits both statistical precision and generalizability. Second, the analytic cohort was further reduced by the availability of pre-colonization stool samples, though clinical characteristics between included and excluded patients were similar (Table S1). Third, antibiotic exposure during ICU stay prior to VRE acquisition and time at risk for acquisition were not fully captured in the primary analysis. Fourth, 16 S rRNA gene sequencing provides less resolution for identifying taxa at the species level than methods such as shotgun sequencing. Despite this, both methods used (SILVA classifier and unassigner) showed concordant results indicating that *C. scindens* is negatively associated with VRE acquisition. Similarly, while imputed metagenomics suggested that *Bai* pathway might be involved in preventing VRE acquisition, the shotgun approach is warranted for validating both species or strain-level associations and metagenomic association. Several additional considerations warrant mention. Patients who acquired VRE had significantly longer ICU stays (median 14 vs. 7 days, *P* = 0.001). Although the time from ICU admission to first VRE-positive screen (median 6 days) was comparable to the ICU stay duration of VRE -/- patients (median 7 days, *P* = 0.052), the longer post-acquisition ICU stay likely reflects both greater illness severity and extended exposure. Because of the small number of acquisition events, we were unable to perform a stable adjusted analysis incorporating ICU time at risk together with other potential confounders. Though we restricted healthy controls to adults aged 35 years or older (*n* = 27, median age 41 years, range 35–68) to reduce age-related confounding of gut microbiome composition, as over half of the available healthy volunteers were younger than 35 and the ICU cohort was predominantly elderly (median 73 years), the healthy controls remained younger than the ICU patients, and this age difference is a limitation of the comparison. Accordingly, these findings should be interpreted as exploratory and hypothesis-generating and require validation in larger, multicenter cohorts.

## Conclusions

In this single-center ICU cohort, VRE acquisition was associated with lower initial *Bacteroides*/*Enterococcus* ratios, lower *C. scindens* abundance, and lower secondary bile acid concentrations. These findings are exploratory and hypothesis-generating. Larger studies with more complete accounting for antibiotic exposure and time at risk are needed to determine whether these microbiome features are independently associated with VRE acquisition and whether they have future biomarker value.

## Supplementary Information

Below is the link to the electronic supplementary material.

Supplementary Material 1.

## Data Availability

The 16 S rRNA gene sequencing datasets generated and/or analysed during the current study are deposited in the National Center for Biotechnology Information Sequence Read Archive under accession number PRJNA1033627.

## Abbreviations

ANCOM-BC: Analysis of compositions of microbiomes with bias correction
ASV: Amplicon sequence variant
Bai: Bile acid-inducible
CI: Confidence interval
DCA: Deoxycholic acid
EC: Entire cohort
GEE: Generalized estimating equations
HSCT: Hematopoietic stem cell transplantation
ICU: Intensive care unit
LCA: Lithocholic acid
PC: Predictive cohort
PCoA: Principal coordinate analysis
VRE: Vancomycin-resistant Enterococcus
