# Supplementary material for: Gut microbiome features associated with vancomycin-resistant Enterococcus acquisition in intensive care unit patients
Source: Ann Clin Microbiol Antimicrob. 2026 Jun 2;25:29. doi: 10.1186/s12941-026-00871-6 (PMC13483550; doi:10.1186/s12941-026-00871-6)
Supplement: Supplementary file 1 [file 12941_2026_871_MOESM1_ESM.docx]

**SUPPLEMENTARY METHODS**

**16S rRNA Gene Sequencing**

The 16S gene V3-V4 region was amplified using 16S forward primer with overhang linker sequence: 5′-TCGTCGGCAGCGTCAGATGTGTATAAGAGACAGCCTACGGGNGGCWGCAG-3′, and 16S reverse primer with overhang linker sequence: 5′-GTCTCGTGGGCTCGGAGATGTGTATAAGAGACAGGACTACHVGGGT ATCTAATCC-3′. PCR reactions were carried out in 25μL reactions with 0.5μL of KAPA® HiFi HotStart Ready Mix (KAPA BIOSYSTEMS), 1 μM of each primer, and 1 ng DNA template. Amplification was performed with 95°C for 3 min, and 25 cycles of denaturation at 95°C for 30 sec, annealing at 57 °C for 30 sec, and elongation at 72 °C for 30 sec, followed by 5 min extension at 72 °C. PCR products were examined on Qsep400 with Standard Cartridge (Bioptic Inc., Taiwan) and a Qubit 2.0 Fluorometer (Thermo Scientific, Waltham, MA, USA). The amplicons were purified by AMPure XP system (Beckman) followed by Nextera XT Index Kit v2 Set (Illumina, San Diego, CA, USA), and the quality of the libraries was assessed on a Qubit 2.0 Fluorometer (Thermo Scientific, Waltham, MA, USA) and Fragment Analyzer 5200 system (Agilent Technologies, CA, USA). Libraries were then equilibrated and sequenced on an Illumina MiSeq platform with 300 bp paired-end reads (generated by Genomics, BioSci & Tech Co., New Taipei City, Taiwan). Paired-end reads (2 × 300 bp) were trimmed by Trimmomatics (version 0.39) and demultiplexed by in-house script.

**Bile acids analysis**

Lyophilized fecal pellets (30 mg) were dissolved in 750 µL of ice-cold water, vortexed using an at 1500 rpm for 5 min, subjected to 20 min of ultrasonication, followed by vortexing again, all at room temperature. Samples were then centrifuged at 18,000 rcf at 4 ℃ for 15 min to obtain supernatants. The remaining pellets were subjected to a second extraction using 750 µL of ice-cold MeOH containing internal standards. Sample were then stored at -20°C. DCA and LCA concentration were then measured using ultra-high-performance liquid chromatography–tandem mass spectrometry.

**Definition of clinical parameters**

Anti-anaerobic antibiotics included penicillins (excluding oxacillin), flomoxef, cefmetazole, cefoxitin, cefoperazone/sulbactam, carbapenems, clindamycin, tigecycline, moxifloxacin and metronidazole. Glycopeptides included vancomycin and teicoplanin. Carbapenems included imipenem/cilastatin, meropenem, doripenem, and ertapenem.

**Supplementary Table S1. Clinical characteristics of VRE-/+ patients included and not included in the predictive cohort (PC)**

| Variable*^a^* | PC  (n = 9) | Not in PC  (n = 14) | *P* |
| --- | --- | --- | --- |
| **Demographics** |  |  |  |
| Age (years) | 66.0 (60.1–79.5) | 77.7 (64.4–81.1) | 0.53 |
| Male | 7 (77.8) | 6 (42.9) | 0.20 |
| Hospital stay ≤ 3 days before ICU admission | 7 (77.8) | 12 (85.7) | 0.99 |
| Duration to first sample (days) | 3 (1–3) | 6.5 (4-7) | 0.02 |
| Nursing home residents | 0 (0) | 0 (0) | n.a. |
| Admission history within 3 months | 2 (22.2) | 7 (50.0) | 0.23 |
| **Underlying condition** |  |  |  |
| Charlson Comorbidity Index | 3 (3–6) | 3 (2–5) | 0.17 |
| Myocardial infarction | 0 (0) | 0 (0) | n.a. |
| Congestive heart failure | 4 (44.4) | 4 (28.6) | 0.66 |
| Cerebrovascular disease | 0 (0) | 0 (0) | n.a. |
| Chronic obstructive pulmonary disease | 1 (11.1) | 1 (7.1) | 0.99 |
| Autoimmune disease | 0 (0) | 0 (0) | n.a. |
| Liver cirrhosis | 0 (0) | 3 (21.4) | 0.25 |
| Diabetes mellitus | 4 (44.4) | 5 (35.7) | 0.99 |
| Chronic kidney disease | 3 (33.3) | 2 (14.3) | 0.34 |
| Renal replacement therapy | 1 (11.1) | 2 (14.3) | 0.99 |
| Malignancy | 7 (77.8) | 7 (50.0) | 0.23 |
| Hematologic malignancy | 2 (22.2) | 1 (7.1) | 0.54 |
| Use of immunosuppressive agents | 8 (88.9) | 12 (85.7) | 0.99 |
| Corticosteroids | 8 (88.9) | 12 (85.7) | 0.99 |
| Chemotherapy | 1 (11.1) | 2 (14.3) | 0.99 |
| **Initial antibiotics at intensive care unit admission** |  |  |  |
| Carbapenems | 3 (33.3) | 1 (7.1) | 0.26 |
| Anti-Pseudomonas Carbapenems | 2 (22.2) | 1 (7.1) | 0.54 |
| Anti-Anaerobics | 8 (88.9) | 13 (92.9) | 0.99 |
| Glycopeptide | 3 (33.3) | 5 (35.7) | 0.99 |
| In-hospital mortality | 6 (66.7) | 5 (35.7) | 0.21 |

*^a^* For continuous variables, median and interquartile range were presented and compared using Wilcoxon rank-sum test. Categorical variables were presented as numbers and percentages and compared using Fisher's exact test.

**Abbreviations:**

PC, predictive cohort; ICU, intensive care unit; n.a., not applicable;

VRE-/+, VRE negative on initial screening and acquired VRE during ICU stay.

**Supplementary Table S2. Antibiotic exposure during ICU stay prior to VRE acquisition or censoring, by VRE colonization group.**

| **Antibiotic class** | **VRE -/- (n=51)** | **VRE -/+ (n=23)** | ***P*** |
| --- | --- | --- | --- |
| Carbapenem | 22 (43.1) | 7 (30.4) | 0.44 |
| Anti-pseudomonal carbapenem | 22 (43.1) | 6 (26.1) | 0.20 |
| Anti-anaerobic agent | 48 (94.1) | 23 (100) | 0.55 |
| Glycopeptide | 22 (43.1) | 13 (56.5) | 0.32 |
| 4th-generation cephalosporin | 11 (21.6) | 5 (21.7) | 1.00 |
| 3rd-generation cephalosporin | 18 (35.3) | 7 (30.4) | 0.79 |

Data are presented as n (%). P values were calculated using Fisher’s exact test. Observation period: ICU admission to first VRE-positive screen (VRE -/+) or ICU discharge (VRE -/-).

**Abbreviations:**
VRE-/-, VRE negative on initial screening and remained negative;
VRE-/+, VRE negative on initial screening and acquired VRE during ICU stay
